# Long-term benefits of exclusive human milk diet in small for gestational age neonates: a systematic review of the literature

**DOI:** 10.1186/s13052-024-01648-3

**Published:** 2024-04-29

**Authors:** Federica Pagano, Emanuele Gaeta, Francesca Morlino, Maria Teresa Riccio, Maurizio Giordano, Giuseppe De Bernardo

**Affiliations:** 1 Department of Translational Medical Sciences, Section of Pediatrics, University of Naples Federico II, Naples, Italy; 2 Department of Clinical Medicine and Surgery, University of Naples Federico II, Naples, Italy; 3 Department of Woman and Child, Ospedale Buon Consiglio Fatebenefratelli, Naples, Italy

**Keywords:** Breastfeeding, Newborn, Nutrition, Donor milk, Outcome, IUGR

## Abstract

Evidence about feeding practices’ consequences in small for gestational age newborns is not well established because they are less likely to initiate and continue breastfeeding than other newborns. Our aim was to study current knowledge about the benefits of exclusive human milk diet after 2 years of age in small for gestational age newborns. A systematic review of the literature was conducted according to Preferred Reporting Items for Systematic Reviews and Meta-Analyses (PRISMA) guideline criteria. Pubmed and Scopus were searched for studies published from databases inception until June 2, 2023. Included articles were analysed and synthesised. Risk of bias and level of evidence assessments were performed. They were enrolled small for gestational age newborns fed by breastfeeding, breast milk or donor milk. The systematic review included 9 articles which were related to 4 health domains: neurodevelopment, cardiovascular, somatic growth and bone mineralization and atopy. Extracted data support a beneficial effect of breastfeeding on these outcomes. Better quality of evidence and longer follow-up are needed.

## Introduction

Breastfeeding, particularly exclusive breastfeeding for the first six months, is widely recognized as the optimal nutrition for infants, endorsed by the World Health Organization (WHO) and United Nations Children’s Fund (UNICEF), who recommend that all children, in particular those belonging to risk categories as low-birth weight for premature infants initiate breastfeeding within the first hour of birth and be exclusively breastfed for the first 6 months of life [1]. Implementation of exclusive breastfeeding in all neonates could improve child development and reduce healthcare costs, resulting in economic savings both for individual families and for countries. Currently, about 44% of infants aged 0–6 months are exclusively breastfed, and lower rates are registered in middle-high income countries. For this reason, WHO actively promotes breastfeeding and is working to increase this rate up to at least 50% by 2025 [2]. WHO estimated that over 820 000 children’s lives would be saved every year among children under 5 years, if they were optimally breastfed [2]. Breastfeeding long-term effects in the general population also include reduction in the odds of overweight, obesity, type 2 diabetes, leukaemia and higher intelligence quotient (IQ), schooling and earnings in adult life [3]. According to WHO, breastfeeding should also be the preferred mode of infant feeding in difficult situations, for instance low-birthweight or premature infants. The Baby Friendly Initiative, championed by UNICEF in 2013, provides evidence that breastfeeding reduces the risk of various neonatal infections and long-term health conditions, including diabetes and cardiovascular disease. Long-term risks for SGA babies who don’t receive human milk include lower cognitive development, increased incidence of infections including gastroenteritis, respiratory and ear infections, higher risk of chronic diseases such as diabetes, hypertension, obesity, and cardiovascular disease among babies [4–7]. In the last two decades, potential changes in the nutritional management of these babies towards implementation of an exclusive human milk diet replacing formula milk were encouraged by experts and scientific societies because of the specific risks of this particular population [8, 9]. Nevertheless, small for gestational age (SGA) neonates are less likely to initiate, establish or continue breastfeeding than the adequate and large for gestational age ones [10]. This disparity has been proven in different socio-economic settings, both in developed and developing countries [11, 12]. Despite the short-term benefits in this population being well known (e.g., increased survival rates, higher probability of successful catch-up growth and lower incidence of complications) [13, 14], the current body of evidence about the long-term outcomes is yet to be established. The aim of our study is to perform a systematic review of the current evidence about the long-term (> 24 months) health benefits deriving from exclusive human milk diet in children born SGA or with intrauterine growth restriction (IUGR).

## Materials and methods

### Methods

This study conformed to the Preferred Reporting Items for Systematic reviews and Meta-Analyses guidelines (PRISMA) [15].

### Eligibility criteria

#### Study designs

Retrospective and prospective observational studies and randomized controlled clinical trials in any language were included in the analyses. Reviews, systematic reviews or commentaries and editorial letters were excluded.

### Patients

Patients’ inclusion criteria were: SGA or IUGR newborns fed by breastfeeding, breast milk or donor milk. SGA were defined as birth weight is below the 10th percentile for their gestational age. IUGR were defined as failure to achieve the full growth potential during gestation, characterized by a slower than expected rate of fetal growth, a birth weight below the 10th percentile for gestational age or a birth weight lower than expected based on other fetal parameters such as abdominal circumference, head circumference, and femur length [16].

Instead, exclusion criteria were: follow-up < 2 years, use of formula milk, not reporting effects related to feeding, not reporting SGA or IUGR infants. Publications in duplicate were removed.

### Information sources and search strategy

Publications were searched in PubMed and Scopus databases from their inception to 2 June, 2023. Databases were screened by two independent researchers. Articles were selected first by titles and then by abstracts. For those with potentially eligible, the full texts were read. Secondary references were screened and included in the analysis, if relevant. Search strategies are shown in Table 1.

**Table 1:** Article search strategy

Database	Number	Search terms
**Pubmed**	S1	((breastfeeding[Title/Abstract]) OR (human milk[Title/Abstract]) OR (breast milk[Title/Abstract]) OR (mother milk[Title/Abstract]) OR (bank milk[Title/Abstract]) OR (donor milk[Title/Abstract])) AND ((SGA[Title/Abstract]) OR (small for gestational age[Title/Abstract]) OR (IUGR[Title/Abstract]) OR (intrauterine growth restriction[Title/Abstract]))
	S2	Breastfeeding (Title) and small for gestational age (Title)
	S3	Breastfeeding (Title) and SGA (Title)
	S4	Breastfeeding (Title) and IUGR (title)
	S5	Breastfeeding (Title) and intrauterine growth restriction (Title)
	S6	Human Milk (Title) and SGA (Title)
	S7	Human Milk (Title) and small for gestational age (Title)
	S8	Human Milk (Title) and IUGR(Title)
	S9	Human Milk (Title) and intrauterine growth restriction (Title)
	S10	Donor milk (Title) and small for gestational age (Title)
	S11	Donor milk (Title) and SGA (Title)
	S12	Donor milk (Title) and IUGR (Title)
	S13	Donor milk (Title) and intrauterine growth restriction (Title)
	S14	Breast pump (all) and SGA (all)
	S15	Breast pump (all) and small for gestational age (all)
	S16	Breast pump (all) and intrauterine growth restriction (all)
	S17	Breast pump (all) and IUGR (all)
	S18	Bank milk (all) and SGA (all)
	S19	Bank milk (all) and small for gestational age (all)
**Scopus**	S20	TITLE-ABS-KEY ( ( breastfeeding OR human AND milk OR donor AND milk OR bank AND milk OR mother AND milk OR breast AND milk ) AND ( SGA OR small AND for AND gestational AND age OR IUGR OR intrauterine AND growth AND restriction ) )

### Selection process

All records were distributed equally among researchers. Endnote X9 (Bld 12,062) was performed to remove duplicates. All researchers screened independently records for title, abstract and the full text was read for eligible manuscripts. In case of disagreement on the inclusion, a meeting was held to obtain the decision. If abstracts or articles required translation into another language to determine their eligibility, google translate was used.

### Data collection process

Data collected were discussed and selected in a meeting. Data collection of the reports were extracted by 4 researchers independently and in duplicate. Any disagreement among data collectors was solved by 2 researchers (MG and GDB).

### Data items

Data were collected in a spreadsheet (Excel 2007) and are: (1) author information, (2) article title, (3) year of publication, (4) study design, (5) exclusion criteria, (6) population, (7) definition of SGA or IUGR, (8) sample size, (9) birthweight, (10) type of milk, 11. types of comparison, 12. outcomes, 13. follow up time, 14. conclusions.

### Study risk of bias assessment

The risk of bias was evaluated by Risk of Bias In Non-randomized Studies - of Interventions (ROBINS-I) or Cochrane Risk of Bias tool (RoB V.2.0) [17, 18]. Two researchers independently assessed the risk of bias of the included publications. Any issue was settled in a meeting.

### Certainty assessment

Grading of Recommendations Assessment, Development and Evaluation (GRADE) approach was used to evaluate the classification of evidence and strength of recommendations. Quality of evidence was rated down by 5 domains (risk of bias, inconsistency, no direct evidence, imprecision, publication bias) or rated up by 3 domains (effect, dose-response, confounding factors) and expressed as 4 levels of evidence: VERY LOW, LOW, MODERATE or HIGH [19].

## Results

### Study selection

**Fig. 1:**
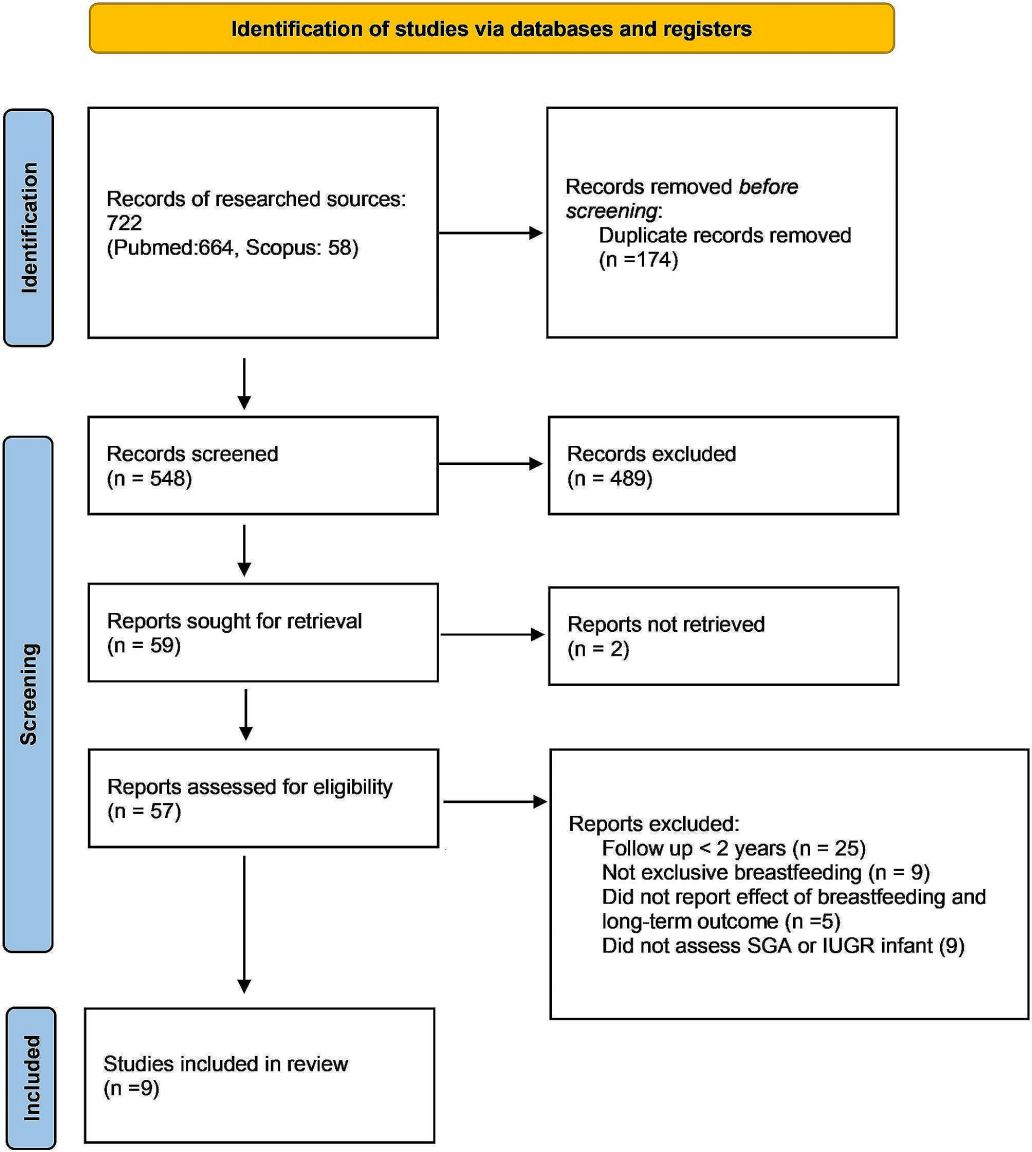
PRISMA 2020 flow diagram for new systematic reviews which included searches of databases and registers only

The study selection process is illustrated in Fig. 1, according to PRISMA 2020 flow diagram for new systematic reviews. We identified 722 studies (664 on PubMed, 58 on Scopus). We merged the two databases and removed 174 duplicates. In the identification phase, 548 records were included based on titles and abstracts, while 489 records were excluded because they did not match the aims of the study. In the screening phase 59 studies were included, while 2 studies were excluded because they were not available in NILDE (Network for Inter-Library Document Exchange). A total of 57 studies were assessed for eligibility by full-text reading and 48 studies were excluded considering the following criteria: follow up < 2 years (*n* = 25), not exclusive breastfeeding (*n* = 9), did not report effect of breastfeeding and long-term outcome (*n* = 5), did not assess SGA or IUGR infant (*n* = 9). Reviews (*n* = 4) and commentary (*n* = 1) were excluded and none of the secondary references were eligible for our study [8, 20–23]. Finally, 9 studies were included in the analysis.

### Study characteristics

General characteristics of the analysed studies were reported in Table 2. The nine studies that were included in the final analysis range from 2002 to 2021 [24–32]. Three studies analysed benefits associated with breastfeeding in SGA and adequate for gestational age (AGA) newborns [25, 28, 31]. Three studies executed a comparison between exclusive breastfed and non-exclusive breastfed SGA newborns [26, 30, 32]. One study compared exclusively breastfed longer than 12 weeks and breastfed for shorter periods SGA newborns [27]. One study performed a comparison between breastfed longer than 6 months and breastfed for shorter periods in foetal growth restriction (FGR) newborns [29]. One study compared SGA newborns who were breastfed or not breastfed during hospitalisation [24]. Exclusively one research studied the use of donor breast milk [32]. There was wide variability in sample size: the smallest study included 30 infants (20 SGA vs. 10 AGA) [28], while Rao et al. conducted the largest one comprising 568 newborns, including 243 SGA newborns [27]. Furthermore, among the 9 selected studies, 7 were prospective cohort studies, 1 was a case-control study, 1 was a randomized controlled trial. All the studies were written in English, except for one which was published in Spanish and English.

**Table 2:** Table of evidence of included studies

Year	Author	Study design	Sample size	Mean (± SD) Birthweight	Milk	Comparison	Age range of outcome (years)	Conclusion	GRADE	Biases and confounding factors
2015	Gibertoni et al. [24]	Cohort study, prospective	316 (54 SGA)	1149.1 g (± 341.2)	HM	Human milk vs. mixed/exclusive formula feeding	0–2	Beneficial effect of breastfeeding for neurodevelopment at 24 months CA and reduced sepsis and MV incidence	LOW	Loss to follow-up (28.6%). Lack of information on duration and dosage of human milk feeding. Lack of information on timing of switching from human to formula feeding. Indirectness.
2013	Savchev et al. [25]	Cohort study, prospective	223 (112 SGA)	2416 g (± 280)	BF	Breastfed SGA vs. breastfed AGA	0–2	Term SGA newborns without signs of placental insufficiency, according to currently used UA Doppler criteria, had lower neurodevelopmental scores	VERY LOW	Differences in neurodevelopmental performance may be clinically irrelevant. Confounding factors (low socioeconomic status was more prevalent in the SGA group).
2005	Slykermann et al. [26]	Cohort study, prospective	550 (223 SGA)	Not reported	BF	Breastfed SGA vs. non breastfed SGA	3–5	In the SGA group, breastfeeding was significantly related to IQ at 3.5 y. SGA children who were breastfed for at least 5 months, had the highest intelligence scores among all the groups.	HIGH	Lack of information about anthropometric parameters that could affect the outcome
2002	Rao et al. [27]	Cohort study, prospective	568 (243 SGA)	BF < 12 wks = 2878 g (± 261) BF > 12 wks = 2866 g (± 222)	BF	Breastfeeding duration (12 vs. 24 weeks)	0–5	Significant impact of exclusive breastfeeding on cognitive development without compromising growth among children born SGA	LOW	Lack of information on indicators reflecting parenting attitudes, home environment and child rearing practices, maternal verbal IQ. Maternal education was not determined for women entered the study on the day of delivery.
2021	Santiago et al. [28]	Cohort study, prospective	32 (20 SGA)	2.370 g	BF	Breastfed SGA vs. breastfed AGA	4–6	In the SGA group, there was no relation between breastfeeding time and increased body fat in any of the analyses performed (BMI, CC, CP, and skinfolds).	VERY LOW	Small sample size due to drop-out. No control group (non-breastfed babies); no detailed analysis about the complementary-fed group. Lack of information about factors that could influence the outcome (e.g., familiar lifestyle or recurrence of metabolic disease). Reporting bias. Indirectness.
2016	Rodriguez- -Lopez et al. [29]	Cohort study, prospective	202 (81 SGA)	1.830 g	BF	Formula feeding vs. breastfeeding (< 1 mo vs. 1–6 mo vs. > 6 mo)	4–5	Positive association between LVSI and cIMT in SGA infants in the groups Breastfeeding > 6 mo	VERY LOW	Small sample size. Lack of information about possible maternal and family-related factors. One time point of BP ignores daily fluctuations.
2022	Vizzari et al. [30]	Cohort study, retrospective	175 SGA	1.770 g	HM	Human milk-fed SGA vs. complementary-fed SGA	0–3	Failure to administer human milk during hospitalization is a risk factor for absence of catch-up growth in both weight and length	LOW	Lack of body composition assessment and analysis of breastfeeding duration to evaluate a possible dose-dependent effect of human milk. Factors that could interfere with feeding decisions (jaundice, hypoglycaemia) were not considered.
2009	Fewtrell et al. [32]	RCT	201 (34 SGA)	1.369 g	HM	Human milk-fed SGA vs. formula-fed SGA	0–20	Subjects receiving > 90% human milk had significantly higher WBBA and BMC than those receiving < 10%	MODERATE	Information detrition due to long-term follow-up. Selection bias. Generalisability.
2005	Purvis et al. [31]	Case-control	550	Not reported	BF	Breastfed SGA vs. breastfed AGA	0–4	Breastfed SGA neonates had higher risk of developing Atopic Dermatitis than those non-Breastfed	LOW	Loss at follow-up. Selection bias. High socioeconomic status was prevalent.

SGA: small for gestational age; AGA: adequate for gestational age; CA: correct age; GA: gestational age; MV: mechanical ventilation; UA: umbilical artery; IQ: Intelligence quotient; BF: breastfed; HM: human milk; BMI: body mass index; CC: cephalic circumference; CP: cephalic perimeter; LVSI: Left ventricular sphericity index; cMIT: carotid intima-media thickness; BP: body pressure; PM: post-menstrual; WBBA: whole body bone area; BMC: bone mineral content; mo: month; RCT: randomized controlled trial

### Risk of bias in studies

ROBINS-I was used to determine the risk of bias for studies and it was reported in Table 2. The Cochrane Risk of Bias tool (RoB V.2.0) was used to determine the risk of bias for randomized controlled trials (Table 2). Level of quality of evidence and studies bias assessment were reported in Table 2.

### Results of individual studies and synthesis of results

The nine studies which fulfilled inclusion criteria were related to four health domains based on literature research: Neurodevelopment, Cardiovascular, Somatic growth and Bone mineralization, Atopy. They were summarized in Tables 2 and 3 according to the same classification. In the Cochrane Handbook for Systematic Reviews of Interventions is stated that meta-analysis should only be executed if studies included are sufficiently homogeneous in terms of participants, interventions and outcomes to provide a meaningful summary [33]. Different topics in our systematic review have been analysed and presented separately but a meta-analysis was not performed because the study groups were not homogeneous and contained some grade of clinical diversity.

**Table 3:** Main results of the studies included in the systematic review

Year	Author	Endpoint	Groups	Results
2015	Gibertoni et al. [24]	Neurodevelopment: GQ evaluated using revised Griffiths Mental Development Scale 0–2 years	HMF vs. FF	Human milk Infants’ GQ higher than formula or mixed milk infants’ GQ (*p* = 0.05)
2013	Savchev et al. [25]	Neurodevelopment: Bayley-III Cognitive Index scores	BF SGA vs. BF AGA	- Cognitive domain scores: AGA > SGA (*p* = 0.027*) - Language domain scores: AGA > SGA (*p* = 0.025*); - Motor domain scores: AGA > SGA (*p* = 0.027*); - Socioemotional domain scores: AGA = SGA (*p* = 0.334); - Adaptive domain scores: AGA > SGA (*p* = 0.012*)
2005	Slykermann et al. [26]	Neurodevelopment: IQ scores using Raven’s Coloured Progressive Matrices	BF SGA vs. NBF SGA	Significant positive correlation between breastfeeding and IQ scores (*p* = 0.03*)
2002	Rao et al. [27]	Neurodevelopment: IQ scores using Wechsler Preschool and Primary Scales of Intelligence—Revised	BF duration (12 vs. 24 weeks)	Long-breastfed SGA’s IQ higher than non-long breastfed SGA’s IQ (*p* < 0.01*)
2021	Santiago et al. [28]	Cardiovascular risk: BMI, CC, CP, skinfolds; Triglycerides, Cholesterol, LDL, HDL, Glycemia, HOMA-IR, SBP, DBP	BF SGA vs. BF AGA	Non-significant difference at 4–6 years for anthropometric (BMI, CC, CP, and skinfolds) by duration of EBF in SGA group; Non-significant difference at 4–6 years in laboratory cardiometabolic parameters between EBF SGA and AGA. (Unreported p-value for BMI, CC, CP, and skinfolds; Triglycerides *p* = 0.921; Cholesterol *p* = 0.921; LDL *p* = 0.795; HDL *p* = 0.399; Glycemia *p* = 0.124; HOMA-IR *p* = 0.072; SBP *p* = 0.064; DBP *p* = 0.306)
2016	Rodriguez-Lopez et al. [29]	Cardiovascular risk: Left ventricular sphericity index (LVSI), carotid intima-media thickness (cMIT), systolic blood pressure (SBP), diastolic blood pressure (DBP)	FF vs. BF (< 1 mo vs. 1–6 mo vs. > 6 mo)	Breastfeeding > 6 mo reduces LVSI and cIMT in SGA infants (*p* = 0.02*) No significant interaction could be observed for breastfeeding an BP (*p* = 0.22)
2022	Vizzari et al. [30]	Somatic growth: Catch up growth in weight and length for the first 3 years	HMF SGA vs. complementary-fed SGA	Failure to administer human milk during hospitalization is a risk factor for absence of catch-up growth in both weight and length (Absence of weight catch-up growth: OR (95% CI) = 0.59 (0.4–0.9) *p* = 0.011*; Absence of length catch-up growth: OR (95% CI) = 0.57 (0.33–0.99) *p* = 0.046*)
2009	Fewtrell et al. [32]	Bone mineralization: WBBA, WBBMC, LSBA, LSBMC measured using DXA	HMF SGA vs. FF SGA	HMF were higher by 5.7% for WBBA, 6% for WBBMC, 8.6% for LSBA and 7.7% for LSBMC (*p* = 0.05). No significant interaction between birthweight category and randomized diet group on later bone outcome
2005	Purvis et al. [31]	Atopy: Prevalence of AD at 3–5 years	Breastfed SGA vs. breastfed AGA	The prevalence was not significantly different between AGA and SGA infants (*p* = 0.52)

*Abbreviations* GQ: General quotient; SGA: small for gestational age; AGA: adequate for gestational age; IQ: intelligence quotient; BMI: body mass index; CC: cephalic circumference; CP: cephalic perimeter; LDL: low density lipoproteins; HDL: high density lipoproteins; HOMA-IR: homeostasis model assessment index; SBP: systolic body pressure; DBP: diastolic body pressure; EBF: exclusive breastfeeding; LVSI: Left ventricular sphericity index; cMIT: carotid intima-media thickness; FGR: foetal growth restriction BP: body pressure; OR: odds ratio; CI: confidence interval; PM: post-menstrual; WBBA: whole body bone area; WBBMC: whole body bone mineral content; LSBA: lumbar spine bone area; LSBMC: lumbar spine bone mineral content; BMC: bone mineral content; AD: atopic dermatitis; BF: breastfed; NBF: non-breastfed; HMF: human milk-fed; FF: formula-fed; mo: month;

*p-value < 0.05

### Neurodevelopment

In 2015, Gibertoni et al. examined the impact of human milk feeding on neurodevelopment at 24 months corrected age in SGA and AGA preterm newborns. The in-hospital feeding protocol prioritised human milk whenever possible. Newborns were fed in 34.5%, 36.1% and 29.4% of the cases by breast milk, mixed milk, or formula milk, respectively. The babies fed by breastfeeding did not take fortified milk. Fortification of bottle-administered human milk was routinely done during hospitalisation at standard dosage with a commercial preparation. Fortification began when enteral intake reached 100 ml/kg and it was interrupted when a body weight of 3.5 kg was achieved. During hospitalisation, it was administered preterm formula milk which contained 80–90 kcal/100 ml and proteins 2–2.3 g/100 ml. After discharge, it was recommended post-discharge formula milk which contained 72–74 Kcal/100 ml and proteins 1.8–1.9 g/100 ml until the weight of 3.5 kg was achieved. The results showed that infants who were fed with human milk scored approximately 3.80 points higher on the Griffiths Mental Development Scale compared to those who received formula milk. This effect remained significant even after adjusting the analysis for complications, growth restriction, and socio-economic status. SGA newborns were associated with a higher probability of complications, including sepsis and the need of mechanical ventilation. They were directly linked to poorer neurodevelopmental outcomes. However, the use of human milk during hospitalisation helped to mitigate the negative effects of lower gestational age and being SGA, resulting in improved neurodevelopmental outcomes (*p* = 0.050). In this study a considerable loss to follow-up (28.6%) was registered. In the analysis there was lack of detailed information about human milk feeding (e.g. duration and dosage) and timing of switching from human to formula feeding. We have identified a significant degree of indirectness in the assessment of bias-related risks [24]. Savchev et al. led a prospective cohort study, the aim was to evaluate the neurodevelopment of SGA vs. AGA term infants in the absence of placental insufficiency. Neurodevelopmental outcome was evaluated at 24 months corrected age using the 3rd edition Bayley Scales of Infant and Toddler Development, which evaluated cognitive, language, motor, social-emotional and adaptive. A total of 223 infants (112 SGA and 111 AGA) were included. The groups differed significantly by socioeconomic status and gestational age at delivery. All neurodevelopmental domains studied were poorer in the SGA group, reaching significance for cognitive (92.9 vs. 100.2, *p* = 0.027), language (94.7 vs. 101, *p* = 0.025), motor (94.2 vs. 100, *p* = 0.027) and adaptive scores (89.2 vs. 96.5, *p* = 0.012). Likewise, the SGA group had a higher risk of low scores in language (OR = 2.63; *p* = 0.045) and adaptive (OR = 2.72; *p* = 0.009) domains. The study showed that breastfeeding did not significantly influence the score in any domain as the percentage of breastfed children was comparable in both groups. Anyway, Bayley Scales could be imprecise as it could underestimate neurodevelopmental disorders in infants or detect differences in neurodevelopmental performance which are clinically irrelevant. Moreover, the lower socioeconomic status of the SGA group was a confounding factor [25]. A study conducted by Slykermann et al. investigated the relationship between breastfeeding and intelligence test scores in 223 versus 308 children who were born SGA and AGA, respectively. Researchers used the Stanford Binet Intelligence Scale. The results demonstrated a significant association between breastfeeding and higher intelligence scores at the age of 3–5 years in SGA children. The study showed that in SGA infants there was a significant positive correlation between average intelligence score and exclusive breastfeeding duration in the univariate (*p* = 0.02) and multivariate (*p* = 0.03) study. In fact, children from the SGA group who were exclusively breastfed for 5 months or longer, had the highest intelligence scores among all the groups (mean = 113.2). However, SGA children were breastfed for shorter periods compared to AGA children. It was not found a difference in intelligence scores between SGA and AGA children. In the analysis anthropometric parameters, which were relevant in this type of population, were not reported (e.g. birthweight) [26]. Rao et al. performed a cohort study to evaluate the effect of exclusive breastfeeding on cognitive development in SGA and AGA term infants, also considering sociodemographic and maternal factors. This study was conducted on a final sample of 519 infants (220 SGA births and 299 AGA). It was assessed IQ at 5 years of age according to the Bayley Scales of Infant Development. This study found that longer duration of exclusive breastfeeding was associated with higher IQ scores at 5 years of age for both groups. The benefits of exclusive breastfeeding on cognitive development were particularly significant for SGA infants. SGA infants exclusively breastfed for more than 12 weeks (mean 109, SD 16) had a significantly higher total IQ compared to those breastfed for shorter periods (mean 100, SD 14; *p* < 0.0001). The study also found a significant association between exclusive breastfeeding duration and factors such as maternal IQ, maternal education, family income, and the child’s attendance at kindergarten. Overall, these findings supported the recommendation to exclusively breastfeed infants for at least 24 weeks to promote cognitive development. In this study, a potential confounding factor was the lack of information about indicators that reflected parenting attitudes, home environment and child-rearing practices. Moreover, maternal education was not determined for enrolled women [27].

### Cardiovascular

In a small sample of breastfed SGA and AGA term babies, Santiago et al. recently evaluated the linkage between the duration of breastfeeding and cardiometabolic parameters until pre-school age. All children were breastfed for at least 6 months, but 36.9% and 41.7% of newborns received complementary feeding in the SGA and AGA group, respectively. It was observed a lower adiposity in the SGA group compared than AGA group at preschool age (percentage of fat in SGA and AGA group, median (interquartile range) = 8.2 (5.7–13.9) and 14.7 (12.01–19.39), respectively; *p* = 0.005). A strong positive correlation was found between body mass index (BMI) at the age of 4–6 years and body fat indicators in SGA group [cephalic perimeter (*r* = 0.7, *p* = 0.001), waist circumference (*r* = 0.6, *p* = 0.002), arm circumference (*r* = 0.9, *p* < 0.001), mid-upper arm muscle area (MUAMA) (*r* = 0.9, *p* < 0.001) and sum of skinfolds (*r* = 0.7, *p* = 0.001)] and in AGA group (cephalic perimeter (*r* = 0.62, *p* = 0.03), cephalic circumference (*r* = 0.9, *p* < 0.001), arm circumference = 0.8, *p* = 0.003) and MUAMA (*r* = 0.6, *p* = 0.03)]. In the SGA, there was no relation between breastfeeding time and BMI, cephalic circumference, cephalic perimeter, and skinfolds, while a strong negative correlation was found in the AGA group between breastfeeding and BMI (*r* = -0.8, *p* = 0.001), cephalic circumference (*r* = -0.7, *p* = 0.007) 0.7, *p* = 0.009), MUAMA (*r* = -0.7, *p* = 0.01). This study had several limitations: the sample size was small and dropout rate during follow-up was high (45% of the initial population), family-related factors that could influence the outcome were not considered in the analysis (e.g. familiar lifestyle of recurrence of metabolic disorders), there was not a control group of non-breastfed babies and there was a lack of details about the complementary-fed group (e.g. duration, proportion of formula milk). Finally, the analysis was not completely reported and part of the object of the present review was evaluated indirectly [28]. In 2016, Rodriguez-Lopez et al. conducted a cohort study that explored the influence of postnatal nutrition on cardiovascular remodelling induced by FGR in comparison with AGA newborns. The two groups were breastfed for a similar duration (median = 4 months, IQR 2–8). The authors confirmed that FGR is the strongest predictor of left ventricular sphericity index (LVSI) (coefficient: −0.4038, 95% CI − 0.4610; −0.3467; *p* < 0.001). Regarding postnatal nutrition, in the overall analysis of the entire sample, only prolonged breastfeeding (> 6 months) showed an independent positive association to LVSI (coefficient: 0.0982, 95% CI 0.0133–0.0183; *p* = 0.02). Breastfeeding was not statistically associated with carotid intima-media thickness (cIMT) and blood pressure (BP). Finally, the combined effect induced by breastfeeding, healthy-fat dietary intake, and overweight/obesity on LVSI and cIMT in FGR children was assessed. It was found that FGR children who were breastfed > 6 months and had a healthy-fat dietary intake showed LVSI and cIMT values closer to AGA newborns. Several data were not considered in the analysis, such as information on potential maternal and family-related factors. Blood pressure was evaluated through a single time point value, which could ignore daily fluctuations [29].

### Somatic growth and bone mineralization

Vizzari et al. in 2022 studied factors associated with failure to perform catch-up growth in a cohort of 175 SGA late-preterm babies. At the time of hospital discharge, the newborns were fed exclusively by human milk, complementary feeding, or exclusive formula milk in 18%, 36% and 46% of the cases, respectively. Enteral feeding was started within the first 24 h of life in all newborns who were in stable clinical conditions. Fresh mother’s milk represented the first choice and mothers were encouraged to directly breastfed their infants or, when this was not possible, to express their milk soon after birth. According to the nutritional procedure of their center, human milk was fortified with bovine milk-based fortifiers in all newborns with weight ≤ 1800 g and with enteral intake ≥ 80 ml/kg/day. Breast milk was more than 50% of the total intake. If breastmilk was insufficient or not available, formula milk feeding was started. It was demonstrated that infants who had not successfully caught-up weight at 12 months were at higher risk of not reaching catch-up growth at 36 months both for weight (OR = 9.31, 95% IC 4.28–20.28; *p* < 0.001) and length (OR = 34.65, 95% IC 11.46-104.77; *p* < 0.001). In this study, logistic regression also demonstrated a significant association between the type of feeding during hospitalisation and the probability of having worse growth trajectories during the entire follow-up: in fact, neonates who did not receive any human milk during hospital stay had lower probability of reaching the 10th percentile for weight (OR = 0.59, 95% IC 0.40–0.9; *p* = 0.011) and length (OR = 0.57, 95% IC 0.33–0.99; *p* = 0.046) at 36 months. Furthermore, this study did not test the association between the type of feeding and head circumference growth in SGA babies. In the current study, it was missing a body composition assessment and analysis of breastfeeding duration to evaluate a possible dose-dependent effect of human milk. Factors that could interfere with feeding decisions (e.g. jaundice, hypoglycaemia) were not considered in the analysis [30]. A randomized trial conducted by Fewtrell et al. in 2009 evaluated the effect of early diet in infants born preterm (< 37 weeks) and birthweight < 1850 g on peak bone mass at 20 years. A total of 201 infants were enrolled, of whom 34 were born SGA, and they were followed-up for 20 years. They were randomized according to the type of feeding in banked donor breast milk (BBM) group or preterm formula (PTF) group. Bone area (BA) and Bone Mineral Content (BMC) were evaluated by Dual X-ray Absorptiometry. At follow-up, BA and BMC were higher in the BBM group by 5.7% for whole body BA, 6% for whole body BMC, 8.6% for lumbar spine BA and 7.7% for lumbar spine BMC (80% statistical power, *p* = 0.05). There were no significant differences in bone turnover markers between groups. Results of the randomized dietary comparisons did not differ between SGA babies below or above 1250 g, and between SGA and AGA babies. There was no significant interaction between the birthweight category and randomized diet group on later bone outcome. In the most vulnerable group of newborns– those born SGA with birthweight < 1250 g– there was no significant differences nor trends in bone outcome measures according to early diet: 7 received BBM (50 kCal/100 mL, 1.3 gr of proteins/100 mL, 33 mg of Calcium/100 mL, 14 mg of Phosphorus/100 mL, without mineral or nutrient fortification), 12 TF (68 kCal/100 mL, 1.45 gr of proteins/100 mL, 35 mg of Calcium/100 mL, 29 mg of Phosphorus/100 mL) and 11 PTF (80 kCal/100 mL, 2 gr of proteins/100 mL, 70 mg of Calcium/100 mL, 35 mg of Phosphorus/100 mL). The authors conducted a punctual analysis of the study biases and limitations, which mainly were selection population bias, generalisability of the results and detrition of information due to long follow-up [32].

### Atopy

A New Zealand case-control study evaluated the prevalence of Atopic Dermatitis (AD) in 3–5 years infants born SGA and AGA. It was discovered that babies breastfed for more than 6 months had an increased risk of AD compared to those not breastfed (OR 9.95, 95% CI 2.66–37.14; *p* = 0.002). Furthermore, no significant difference in the AD prevalence between AGA and SGA infants was reported (16,7% and 14.7%, respectively; *p* = 0.52). The study highlighted how breastfeeding did not worsen atopy outcome in infants born SGA, in fact they had equivalent risk as breastfed AGA. Even if it was not found any association between low birthweight and AD at 3–5 years, it could not be distinguished whether this phenomenon could be observed only for some forms of atopic disease or whether it appeared after the follow-up time. The main limitation of this research was dropout rate of subjects during the follow-up and a homogeneous population (mostly New-Zealand Europeans), with a high mean socioeconomic status [31].

## Discussion

### Challenges in research field

Breastfeeding management is more challenging in SGA than in AGA newborns, for both parents and health workers. Concerns about weight gain in infants are known to often lead to the introduction of formula milk into their diet. In addition, parental factors such as guilt, anxiety, and stress deriving from the need for frequent medical attention also impact on the choice of feeding practices [34, 35]. In 2020, Dooks et al. discovered that mothers of full-term SGA babies were less likely to breastfeed their babies than other mothers. SGA babies were less likely to be exclusively breastfed than non SGA babies both at the time of hospital discharge and discharge from the care of the community midwife. Overall, SGA babies were less likely to receive any type of breastfeeding (both exclusive and mixed) than non SGA babies at the time of community discharge [10]. Our study also highlights a gap in the knowledge about long term outcomes in SGA newborns and provides additional evidence to support the benefits of human breastfeeding in SGA infants as well. For this reason, it is important that evidence-based medicine focuses on the long-term benefits of breastfeeding providing additional support to health care professionals and the community to minimise unnecessary use of formula milk.

### State of the evidence

In the neurodevelopmental domain, most of the data extracted supported the evidence that breastfeeding improved SGA neonates’ cognitive performance, despite heterogeneity in methods and parameters (Griffiths Mental Development Scale, Raven’s Coloured Progressive Matrices, Wechsler Preschool and Primary Scales of Intelligence). Furthermore, in three studies there was an association between higher IQ scores and breastfeeding in SGA babies, also considering the duration of intervention [24–27]. Two of these studies demonstrated how cognitive development of SGA infants was particularly supported by breastfeeding. Slykerman RF et al. compared breastfed versus non-breastfed SGA newborns while Rao MR et al. compared SGA babies breastfed for longer versus shorter periods [26, 27]. However, Savchev et al. showed that the breastfed AGA population had better neurodevelopmental scores than breastfed SGA [25]. The level of evidence of this research was considered very low due to indirectness and confounding bias. The included studies in the cardiovascular domain focused on the effects of different times of breastfeeding. It was revealed that breastfeeding time reduces significantly ventricular remodelling parameters [29]. Cardiometabolic laboratory findings and anthropometric parameters were non significantly different between breastfed SGA and AGA newborns [28]. An interesting piece of evidence researched in the literature was the evaluation of the long-term effects of the type of diet established during hospitalisation and after discharge. Vizzari et al. showed how breastfeeding absence during hospitalisation was an independent risk factor for not performing catch-up growth in a population entirely composed by SGA infants [30]. In this type of population, breastfeeding promotion should be strongly supported starting from the hospital stay. These results were consistent with the research conducted by Dooks et al. who highlighted that SGA neonates who were not breastfed in hospital were less likely to be breastfed in the community compared to non SGA babies [10]. Regarding the evidence about bone mineralization, SGA and AGA newborns fed by human milk showed higher BA and BMC compared to those fed by formula milk [32]. This was the only long-term randomized clinical trial founded but the level of evidence was rated as moderate due to small sample size, indirectness, risk of bias in the randomization process and some concerns about missing data. Moreover, this was the only study that used donor milk. It was demonstrated that donor milk provides the nutritional and immunologic benefits of breast milk and reduce complications (such as infectious diseases, necrotizing enterocolitis, sepsis, bronchopulmonary dysplasia) in preterm or low birthweight infants compared with formula milk, supporting its further implementation. The presence of milk banking was also associated with higher rates of breastfeeding after hospital discharge, even for infants who were admitted to neonatal intensive care units. In a study of 83 neonatal intensive care units in Italy, the prevalence at discharge of exclusive breastfeeding in the units with a human milk bank was 13.9% higher than in those without [36]. The major limitations to use donor milk are the operating cost and, in some settings, societal norms relating to milk sharing [37–39]. Concerning atopic dermatitis, only 1 research revealed an increased risk of atopy in breastfed AGA and SGA infants [31]. The authors suggested that this effect could depend on the maternal risk factors for atopy, maternal diet, complementary feeding introduction time, and high socio-economic status. Similarly, other studies about the association between breastfeeding and atopic disease produced ambiguous results, potentially depending on the population examined, population lifestyle, socioeconomic status, such as mother’s diet during breastfeeding [40–43].

#### Gaps and limitations in knowledge

Our research on the state of art on breastfeeding for SGA neonates and its long-term benefits revealed several major issues in this research field. A great variability in the definitions was found. Among the analysed studies, for example, Rodriguez-Lopez et al. defined FGR as birthweight < 10th percentile, which is commonly used as the definition of SGA [29]. Vizzari et al. similarly defined catch-up growth as achievement of > 10th percentile for weight and/or length by, while the definition used by Santiago et al. was “a difference of ≥ 0.67 standard deviations in the weight/age indicator (W/A) between two moments in a series of observations” [28, 30]. In some of the included studies, the definition of SGA was stated as birthweight < 2 standard deviations below the mean for age and sex, while the most accepted definition was birthweight < 10th percentile for age and sex. Moreover, the same outcome was assessed with different methods by researchers. For instance, neurodevelopment, whose domain belong 4 of our studies, was estimated in different ways in the same age range [24–27]. In the studies that followed-up infants until the 2 years of age, neurodevelopment was evaluated through general quotient by Griffiths Mental Development Scale and Bayley-III Cognitive Index scores [24, 25]. In a similar way studies, which continued follow-up until the 5 years of age also differed for the effect measures. IQ was evaluated according to Raven’s Coloured Progressive Matrices or Wechsler Preschool and Primary Scales of Intelligence [26, 27]. The included studies were not homogeneous in terms of participants, interventions, and outcomes enough to provide a meaningful summary, furthermore few large samples were enrolled. For these reasons, results were not consistent with performing a meta-analysis. The quality evidence of the analysed studies was low, due to study designs and numerous biases. A matter in planning randomized controlled trials on this topic, was to assign neonates and infants to a formula-fed control group, which would be unethical. In addition, regarding the length of follow-up, no long-term follow-up study would reflect current dietary practices as they change dramatically within a few years. For the reason above mentioned, it is needed to conduct multiple studies sharing the same protocols, in order to make the results more comparable. Some gaps in the research about this topic were also identified. Few studies considered the factors that could affect breastfeeding’s outcomes in a detailed way. For instance, they were identified among these factors, the duration of human milk diet and quantity of human milk administered (when donor banked milk of breast pump were used). Only Rodriguez-Lopez et al. conducted an analysis with both a control group (formula-fed infants) and different duration of breastfeeding (< 1 month, 1–6 months, > 6 months) to establish possible duration-dependent effect [29]. Factors that have not been analysed by many researchers were those related to the family (e.g. family socioeconomic status, dietary attitudes, maternal literacy and IQ). Rao et al. found significant associations between exclusive breastfeeding duration and factors as maternal IQ and education, family income and child’s attendance at kindergarten [27]. According to these results, those should be considered in studies on long-term effects of breastfeeding (e.g., to perform multivariate analysis), as they could act as confounding factors. Three studies included preterm newborns in their cohorts [24, 30, 32]. Enrolled newborns had an increased risk of extrauterine growth restriction compared to those born at term. However, researchers did not compare preterm versus term newborns and explored three different research domains (neurodevelopment, somatic growth and bone mineralization). Furthermore, they were evaluated SGA and AGA newborns with the same gestational age category.

#### Our study limitations

The emerging body of evidence surely suffered from lack of high quality studies and shortage of pieces of research about this topic in general. Indeed, the results of the 9 studies reviewed were not supported by large sample size and long follow-up, nor by robust study designs in most cases. In addition, in order to include as many studies as possible, in some cases the outcomes were evaluated indirectly. Indirectness was indicated as bias in the quality of evidence assessment. For example, several studies compared breastfed SGA and AGA newborns, instead of making comparisons between breastfed and non-breastfed SGA newborns. In these cases, when differences between AGA and SGA newborns were not detected, it was assumed that the outcome for the breastfed SGA newborns was effective in filling the differences towards AGA newborns.

## Conclusions

Our research on the current evidence about the long-term benefits of an exclusive human milk diet in SGA and IUGR neonates showed a positive impact on almost all health domains resulting from our analysis. Indeed, a positive effect was found on intelligence quotient and cognitive indexes, anthropometric and cardiometabolic parameters, growth, and bone mineralization. The only exception was atopy, for which babies breastfed more than 6 months had an increased risk of atopic dermatitis. A relevant issue we found during our investigation was the lack of comparability among different pieces of research, even when the same outcome was being investigated. Considering the poor quality of evidence and the shortage of follow-up until the adult age, we would recommend making further investigations in this novel research field in order to enhance the body of evidence with more robust evidence that could bring significant progress in perinatal medicine and public health.

## Data Availability

All data generated or analysed during this study are included in this published article.

## Abbreviations

WHO: World Health Organization
UNICEF: United Nations Children’s Fund
IQ: intelligence quotient
SGA: small for gestational age
IUGR: intrauterine growth restriction
PRISMA: Preferred Reporting Items for Systematic reviews and Meta-Analyses guidelines
ROBINS-I: Risk of Bias In Non-randomized Studies - of Interventions
GRADE: Grading of Recommendations Assessment, Development and Evaluation
NILDE: Network for Inter-Library Document Exchange
AGA: adequate for gestational age
FGR: foetal growth restriction
LVSI: left ventricular sphericity index
cIMT: carotid intima-media thickness
BBM: banked donor breast milk
PTF: preterm formula
BA: Bone area
BMC: Bone Mineral Content
AD: Atopic Dermatitis
